# Remote ischemic preconditioning triggers changes in autonomic nervous system activity: implications for cardioprotection

**DOI:** 10.14814/phy2.13085

**Published:** 2017-02-13

**Authors:** Safa Abdul‐Ghani, Arnold N. Fleishman, Igor Khaliulin, Marco Meloni, Gianni D. Angelini, M‐Saadeh Suleiman

**Affiliations:** ^1^ Bristol Heart InstituteSchool of Clinical SciencesFaculty of Medicine & DentistryUniversity of BristolBristolUnited Kingdom; ^2^ Research Institute for Complex Problems of Hygiene and Occupational DiseasesNovokuznetskKemerovo OblastRussia; ^3^ BHF Centre for Cardiovascular ScienceUniversity of EdinburghEdinburghUnited Kingdom; ^4^ Present address: Department of Physiology & PharmacologyFaculty of MedicineAl‐Quds UniversityAbu‐DisP O Box 89Jerusalem

**Keywords:** Autonomic nervous system, blood flow, cardiac, ischemia, remote ischemic preconditioning

## Abstract

Cardioprotective efficacy of remote ischemic preconditioning (RIPC) remains controversial. Experimental studies investigating RIPC have largely monitored cardiovascular changes during index ischemia and reperfusion with little work investigating changes during RIPC application. This work aims to identify cardiovascular changes associated with autonomic nervous system (ANS) activity during RIPC and prior to index ischemia. RIPC was induced in anesthetized male C57/Bl6 mice by four cycles of 5 min of hindlimb ischemia using inflated cuff (200 mmHg) followed by 5 min reperfusion. Electrocardiography (ECG) and microcirculatory blood flow in both hindlimbs were recorded throughout RIPC protocol. Heart rate variability (HRV) analysis was performed using ECG data. Hearts extracted at the end of RIPC protocol were used either for measurement of myocardial metabolites using high‐performance liquid chromatography or for Langendorff perfusion to monitor function and injury during 30 min index ischemia and 2 h reperfusion. Isolated‐perfused hearts from RIPC animals had significantly less infarct size after index ischemia and reperfusion (34 ± 5% vs. 59 ± 7%; mean ± SE *P* < 0.05). RIPC protocol was associated with increased heart rate measured both in ex vivo and in vivo. Frequency ratio of HRV spectra was altered in RIPC compared to control. RIPC was associated with a standard hyperemic response in the cuffed‐limb but there was a sustained reduction in blood flow in the uncuffed contralateral limb. RIPC hearts (prior to index ischemia) had significantly lower phosphorylation potential and energy charge compared to the control group. In conclusion, RIPC is associated with changes in ANS activity (heart rate, blood flow, HRV) and mild myocardial ischemic stress that would contribute to cardioprotection.

## Introduction

Remote ischemic preconditioning (RIPC) is a phenomenon where preconditioning a tissue distant to the heart confers cardioprotection against index ischemia and reperfusion. It is suggested that the signal from the distant tissue is transmitted to the heart via neural or humoral signaling pathways or an overlap between both (Gho et al. 1996; Gill et al. 2015; Heusch et al. 2015). However, the role of either pathway or the exact mechanism(s) involved is not presently known.

The neural hypothesis proposes that substances (e.g., autacoids, adenosine, and bradykinin) released locally in the remotely ischemic area activate a neural afferent pathway which in turn activates various efferent pathways in the heart that induce cardioprotection (Tang et al. 1999; Schoemaker and van Heijningen 2000; Xiao et al. 2001; Brzozowski et al. 2004a; Dong et al. 2004; Donato et al. 2013). The efferent pathway appears to involve the autonomous nervous system (ANS). Evidence for the involvement of the ANS comes from work showing pretreatment with the ganglionic blocker hexamethonium, inhibits RIPC cardioprotection (Gho et al. 1996). Additionally, the cardioprotective effect of RIPC disappears in animals subjected to nerve resection, vagotomy (Mastitskaya et al. 2016), blockade of muscarinic cholinergic receptors (Ding et al. 2001; Brzozowski et al. 2004b; Dong et al. 2004; Lim et al. 2010; Donato et al. 2013), or silencing vagal preganglionic neurons in the brainstem (Mastitskaya et al. 2012). Recent evidence suggests that cardioprotection is partially mediated by intrinsic cardiac ganglia in response to increased systemic efferent vagal tone (Pickard et al. 2016). Moreover, there is also evidence supporting a role for sympathetic adrenergic stimulation in RIPC‐induced cardioprotection (Taliyan et al. 2010). Involvement of ANS in RIPC‐induced effects also includes changes in organ blood flow and cardiac output. For example, RIPC application in anesthetized pigs was associated with coronary vasodilation in paced hearts (Shimizu et al. 2007). RIPC in conscious volunteers also induces vasodilatation of the contralateral artery (right brachial artery) and improved parasympathetic activity as measured using heart rate variability (HRV) (Enko et al. 2011). However, a recent study could only detect HRV changes in patients with coronary disease but not in healthy volunteers (Zagidullin et al. 2016). Moreover, RIPC in healthy volunteers attenuated I/R sympathetic activation following ischemia/reperfusion limb injury (Lambert et al. 2016). It is evident therefore that the effect of RIPC application on the ANS activity remains controversial. Furthermore, it is anticipated that changes in sympathetic activity would alter cardiac metabolic demand. Direct evidence to support this view is currently lacking as most studies have largely reported changes in signaling pathways after reperfusion (Wolfrum et al. 2002; Yamamura et al. 2005; Serejo et al. 2007; Tamareille et al. 2011). Therefore, the aim of this work was to address these issues and directly link them to cardioprotection using anesthetized mouse model. In particular, we investigated the effect of RIPC on key cardiovascular activities associated with ANS including blood flow, HR, HRV, and myocardial energetics prior to index ischemia.

## Materials and Methods

### Animals and ethics statement

Male C57/BL6 wild‐type mice (25–28 weeks old, 26–33 g, *n* = 60) were used for all experiments. All animals were purchased from B&K Universal. Animals were kept at the University of Bristol Veterinary School until used. Animal work was performed in accordance with the UK Animals (Scientific Procedures) Act of 1986 and approved by the University of Bristol Animal Welfare and Ethical Review Board (Permit numbers: PPL 30/2859 and PIL 30/9564).

### RIPC protocol

**Figure 1:**
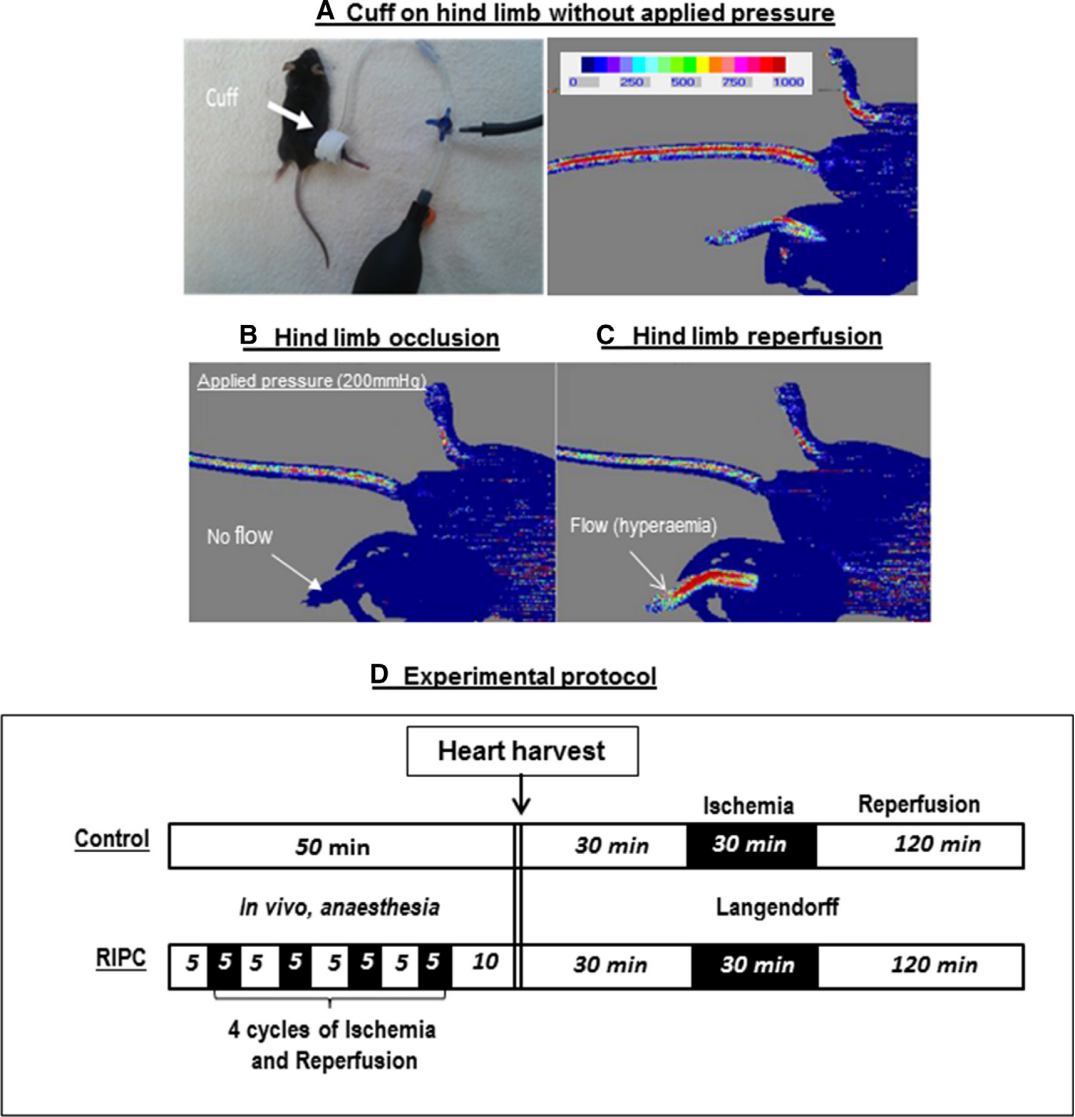
Monitoring microcirculatory blood flow using laser Doppler flowmetry. This figure shows a photo of a mouse with a cuff placed around the hind limb and an image showing both the color intensity scale and intensity in the limbs reflecting blood flow (A). The lower panels show cessation of blood flow during ischemia (200 mmHg cuff inflation) and hyperemia during reperfusion following cuff deflation (B and C). (D) A schematic of the experimental protocols. In summary, mice were anesthetized for 50 min and divided into control and RIPC groups. In the RIPC group, the animals were exposed to four cycles of 5 min limb ischemia followed by 5 min reperfusion. Blood flow in both limbs or ECG was monitored during this period. At the end of RIPC/control protocol, hearts were extracted and used for either metabolite extraction or for Langendorff perfusion. RIPC, remote ischemic preconditioning; ECG, electrocardiography.

Mice were anesthetized by an intraperitoneal injection (0.020 mL/g weight) of 2.5% tribromoethanol (Avertin^®^) (Sigma‐Aldrich, Gillingham, Dorset, U.K.), allowed ~5 min to become fully anesthetized (evidenced by lack of response to toe or tail pinch). A specially designed small (1.6 × 9 cm) pressure cuff (Hokanson, Inc., Bellevue, WA, USA) was placed around the hind limb at the inguinal level (Fig. 1A) and RIPC was induced by four cycles of 5 min of limb ischemia at 200 mmHg followed by 5 min of reperfusion. The applied pressure was selected as it was used earlier (Li et al. 2011) and was associated with complete block of microcirculatory blood flow (Fig. 1B) followed by significant hyperemia (Fig. 1C). Control group had a deflated cuff placed on the lower limb. Body temperature was maintained at around 37°C using a heating pad.

### Langendorff‐perfused heart

At the end of 50 min anesthesia involving control or RIPC protocols, animals were killed by cervical dislocation. Hearts were rapidly excised and placed in cold Krebs–Henseleit (K‐H) buffer. The excess tissue was removed from the heart, and the aorta was cannulated with a metal cannula via modified Langendorff perfusion apparatus and perfused under constant pressure of 60 mmHg with 37°C K‐H solution containing (mmol/L) NaCl 120, KCl 4.8, CaCl_2_ 2, MgSO_4_ 1.2, KH_2_PO_4_ 1.2, NaHCO_3_ 25, and glucose 11. The solution was filtered and continuously gassed with 95% O_2_, 5% CO_2_ (pH 7.4). The perfusion chamber was tightly closed and temperature controlled to ensure the heart was maintained at constant temperature (~37°C) throughout. Cardiac contractile function was measured with a force‐transducer (MLT0402 [2 g]; AD Instruments, Castle Hill, Australia) connected to the apex of the heart using a metal hook. Hearts were preloaded with a resting tension of 250 mg. Perfused hearts were left to stabilize for ~30 min followed by 30 min global ischemia (introduced by switching off the pump) and 2 h reperfusion. Developed tension was monitored using Mac Lab data acquisition system (AD Instruments) and was used to compute heart rate. Cardiac pump function was expressed as rate tension product (RTP), the product of heart rate and developed tension. Developed tension and subsequently heart rate could not be computed in the reperfused injured (hyper‐contracted) heart. Therefore only the following parameters were measured: time to stop beating, time to rigor, changes in diastolic tension and flow rate. Diastolic tension was used as a marker of functional impairment during reperfusion.

### Measurement of infarct size

At the end of reperfusion hearts were perfused in Langendorff mode with 1% (W/V) Triphenyltetrazolium chloride in phosphate buffer saline for 8 min at a flow rate of 2.5 mL/min. After staining, the heart was removed, wrapped in Clingfilm and stored in freezer for 1 h. The frozen heart was cut transversely into four (~2‐mm‐thick) slices from apex to the top of the ventricles and left overnight in 10% formaldehyde for fixation. The viable myocardium stained red, and infarct tissues appeared pale white. Later each side of the slices was scanned by electronic scanner (Epson perfection 4990 photo). The infarct size was determined by computerized planimetry using AlphaEaseFC software (Alpha Innotech, San Leandro, CA, USA). Since the entire heart was at risk from global ischemia, the ratio of necrotic areas to total slice areas of the four myocardial slices were used to estimate percentage of necrosis.

### Measurement of creatine kinase

In all Langendorff experiments involving index ischemia and reperfusion, coronary effluent was collected at regular intervals. Collections were made preischemia, and at 1, 3, 5, 10, 20, 30, 60, 90, and 120 min during reperfusion. Creatine kinase (CK‐MB) activity in these samples was determined using a commercially available kit (Randox, Crumlin, County Antrim, U.K.) and measured using a spectrophotometer at 340 nm, 37°C and expressed as U/L.

### Laser Doppler flowmetry

Blood flow in the hind limb was monitored throughout the RIPC protocol using laser Doppler flowmetry (moorLDI2 imager Perimed, Sweden, and Moor, USA). A color‐coded image representing the microvascular blood flow distribution after each RIPC cycle (after and during 5 min occlusion) for the whole procedure (50 min) was captured on the monitor (see Results below). Color intensity scheme (Fig. 1A) is used to quantify blood flow in the tissue.

### Electrocardiography (ECG) in vivo

Approximately 5 min after anesthetic induction, three needle (29G) electrodes (MLA1204; AD Instruments) were inserted subcutaneously into the right pelvic limb area and into each thoracic limb area of 32 mice (*n* = 19 for control and 13 for RIPC). The ECG signal was recorded and analyzed using Bio amplifier connected to the data acquisition system (Power Lab/8SP; AD Instruments). The data were recorded continuously for the whole RIPC procedure (50 min). Heart rate was estimated using R‐R interval of the least noisy traces of the ECG.

### HRV analysis

HRV is widely used as a measure of ANS activity to the heart (sympatho‐vagal balance) (Roy and Ghatak 2013) and can help to elucidate the role and the contribution of the ANS in RIPC. The HRV and HR analysis was performed using randomly selected ECG recordings from 12 mice (6/group) that had least noisy traces and sufficient signal to enable HRV analysis. The following ranges of the analyzed HRV spectrum were selected for the frequency quantification (Fleishman et al. 2014): low frequency (LF, 0.08–0.12 Hz) and high frequency (HF, 0.15–0.35 Hz). The HF reflects peripheral activity of parasympathetic system while LF predominantly corresponds to the activity of peripheral sympathetic modulations (Task Force of the European Society of Cardiology the North American Society of Pacing Electrophysiology, 1996). Additionally, the ratio (HF/LF) has been used to assess changes in vagus activity relative to the activity of sympathetic system.

### Measurement of cellular metabolites

Cardiac metabolites from hearts collected after 50 min of anesthesia with or without RIPC were extracted and measured using high‐performance liquid chromatography (HPLC) as described previously (Imura et al. 2001; Modi et al. 2004) and based on earlier work (Ally and Park 1992). In brief, frozen myocardial tissue samples were crushed in liquid nitrogen and a small amount added to tube containing 500 *μ*L of 4.8% perchloric acid, vortexed, weighed (to obtain sample wet weight) and then spun at 4500 rpm for 15 min at 4°C. The supernatant was neutralized with equal volume of 0.44 mol/L K_2_CO_3_, vortexed and then spun at 4500 rpm for 15 min at 4°C. The supernatant was taken and stored at −20°C until analyzed for cellular metabolites using an HPLC Beckman System and a 3‐*μ*m octadecyl silane Hypersil column (150 × 4.6 mm) (Thermo Scientific, Newport, U.K.). Eluent A contained 150 mmol/L KH_2_PO_4_ and 150 mmol/L KCl, set at pH 6.0 with KOH. Eluent B consisted of eluent A with 15% (v/v) HPLC grade acetonitrile. A low‐pressure gradient mixing device was used to control the composition of the mobile phase. The amount of eluent B changed linearly between the time points. The analytical column was maintained at a constant temperature in the range 17–19°C and absorption was measured at 254 nm. The metabolites measured were ATP, ADP, AMP, GTP, GDP, GMP, IMP, xanthine, hypoxanthine, Nicotinamide adenine dinucleotide (NAD^+^), inosine, and adenosine. The area under the peak for each metabolite was determined and compared to the standard sample, then corrected for weight.

### Statistical analysis

Statistical analyses were performed using Statveiw for Windows (SAS Institute Inc., Cary, NC, USA) and Prism 5 Version 5.01 software (GraphPad, La Jolla, CA, USA). Data were expressed as the mean ± SEM. Differences between control and RIPC groups were analyzed using unpaired *t*‐test *P* < 0.05 considered significant, whereas differences within the same group were analyzed using paired *t*‐test. Data were tested for normal distribution using Kolmogorov–Smirnov test and equal variance using the *F*‐test. Area under the curve to estimate total creatine kinase release was calculated using Trapezium rule on Excel spread sheet. Repeated measures analysis of variance (ANOVA) was used for changes overtime and significance tested using Bonferroni‐Dunn post hoc test.

## Results

### The effect of RIPC on cardiac injury following index ischemia and reperfusion

**Figure 2:**
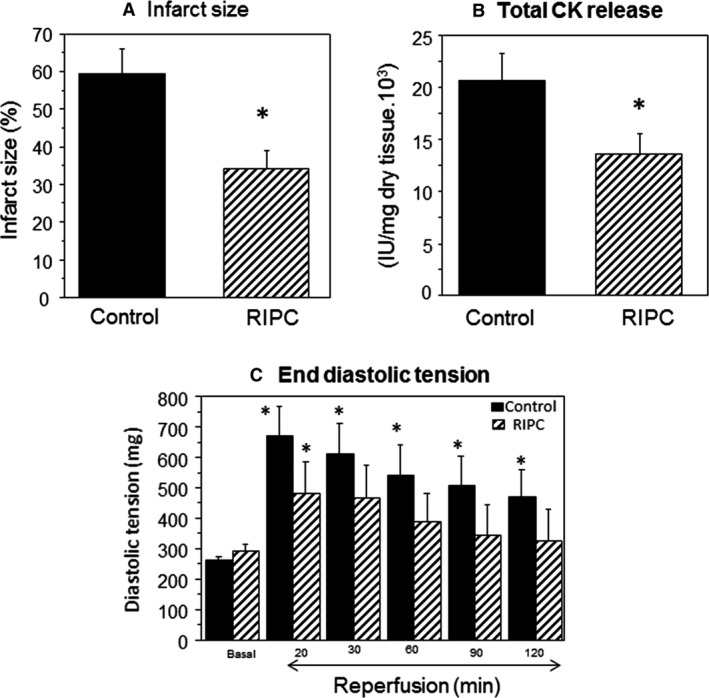
Cardiac ischemia and reperfusion injury in Langendorff heart. The Effects of RIPC on infarct size and creatine kinase release. Langendorff‐perfused hearts with or without RIPC application were exposed to 30 min ischemia followed by 120 min reperfusion. (A) Infarct size measured using TTC and expressed as percentage of risk area. Inset shows representative sections from control and RIPC hearts with darker parts indicating viable tissue. (B) Creatine kinase activity from hearts effluents collected preischemia and during reperfusion. (C) The effects of RIPC on diastolic tension measured at 20, 30, 60, 90, and 120 min during reperfusion. Data are presented as mean ± SEM (*n* = 6–10 hearts/group for different endpoints). *P <* *0.05 versus corresponding control (or basal) using either unpaired *t*‐test (A and B) or repeated measures ANOVA with Bonferroni‐Dunn test (C). RIPC, remote ischemic preconditioning; TTC, triphenyltetrazolium chloride; ANOVA, analysis of variance.

Myocardial injury following index ischemia and reperfusion was determined by measuring release of cardiac enzyme creatine kinase and the infarcted area in control and RIPC hearts (Fig. 2A and B). RIPC induced significant (*P* < 0.05) reduction in the percentage of infarct size compared to control (34 ± 5% vs. 59 ± 7%). RIPC also significantly reduced total release of creatine kinase compared to control (13,000 ± 1000 IU/mg vs. 21,000 ± 2000 IU/mg dry tissue). Furthermore, RIPC was associated with significantly better recovery in diastolic tension measured at different time points during reperfusion compared to control (Fig. 2C). Table 1 shows no effects on parameters during index ischemia (e.g., time to stop beating, time to rigor, changes in diastolic tension).

**Table 1:** Effect of RIPC on cardiac function during index ischemia

	Control	RIPC	*P*‐value
Time to stop beating (min)	8.6 ± 1.2	10.3 ± 4.0	0.66
Time to start rigor contracture (min)	17 ± 2.6	19.3 ± 2.7	0.55
Time to reach maximal diastolic tension (min) after onset of rigor	11.0 ± 2.5	9.3 ± 2.5	0.65
Maximum diastolic tension (mg)	869 ± 105	711 ± 100	0.31

Data are presented as mean ± SEM (*n* = 7–8 hearts/group). RIPC, remote ischemic preconditioning.

### Cardiovascular effects of RIPC prior to index ischemia

#### Effect of RIPC on heart rate in vitro and in vivo

**Figure 3:**
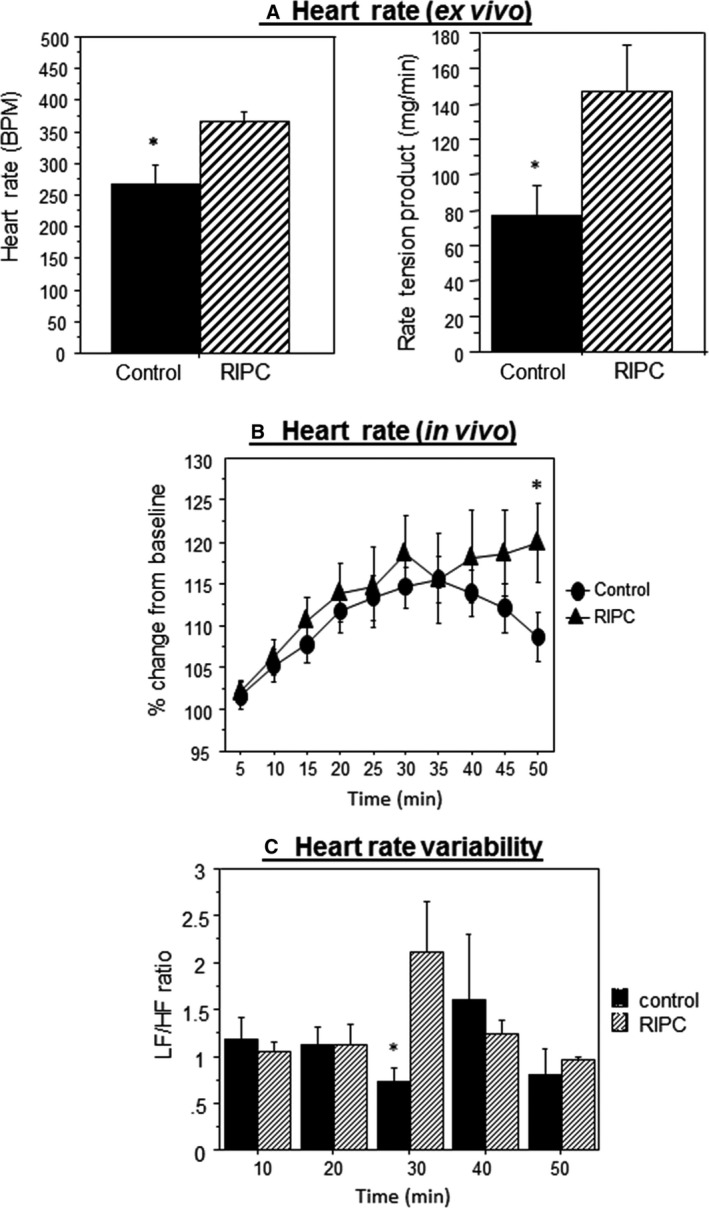
The effect of remote ischemic preconditioning (RIPC) on heart rate and heart rate variability. (A) Changes in heart rate (left panel) and in tension (right panel) prior to ischemia and during equilibration measured in vitro (*n* = 10 hearts control, *n* = 7 hearts RIPC group). (B) Heart rate percentage change from baseline during RIPC protocol in vivo (*n* = 19 for control and *n* = 13 hearts for RIPC group). (C) Low‐/high‐frequency power spectrum density ratio in RIPC and control group during each reperfusion cycle (10, 20, 30, 40, and 50 min). Data are presented as mean ± SEM (*n* = 6 hearts in each group). *P <* *0.05 versus control.

Figure 3A shows the data for computed heart rate and heart RTP measured after 30 min of Langendorff perfusion (prior to index ischemia). Perfused hearts from animals treated with RIPC had a significantly higher heart rate and RTP compared to control hearts.

Following the induction of anesthesia, heart rate drops to levels of around 450 BPM and slowly recovers to higher levels but remains relatively lower than physiological levels of 550–600 (data not shown). The extent of this increase tended to be higher in RIPC compared to control. The percentage change in heart rate from baseline was calculated for each mouse in vivo. There was a gradual and similar increase in heart rate for both control and RIPC groups for most of the anesthesia duration except for the period 30–35 min (Fig. 3B). However, the percentage in HR started to decline in control group but remained high in RIPC group during the remaining period of RIPC. At the end of the 50 min duration there was a significant difference between the two groups. The data for changes in the ratio between low and HF components of HRV spectra (LF/HF ratio) using HRV analysis, represent a measure of balance of sympatho‐vagal activity. Up to 20 min of RIPC, there was no difference between LF/HF ratios compared to control (Fig. 3C). However, at 30 min the ratio was higher for RIPC but this was not sustained toward the end of RIPC protocol.

#### The effect of RIPC on cardiac metabolites

**Figure 4:**
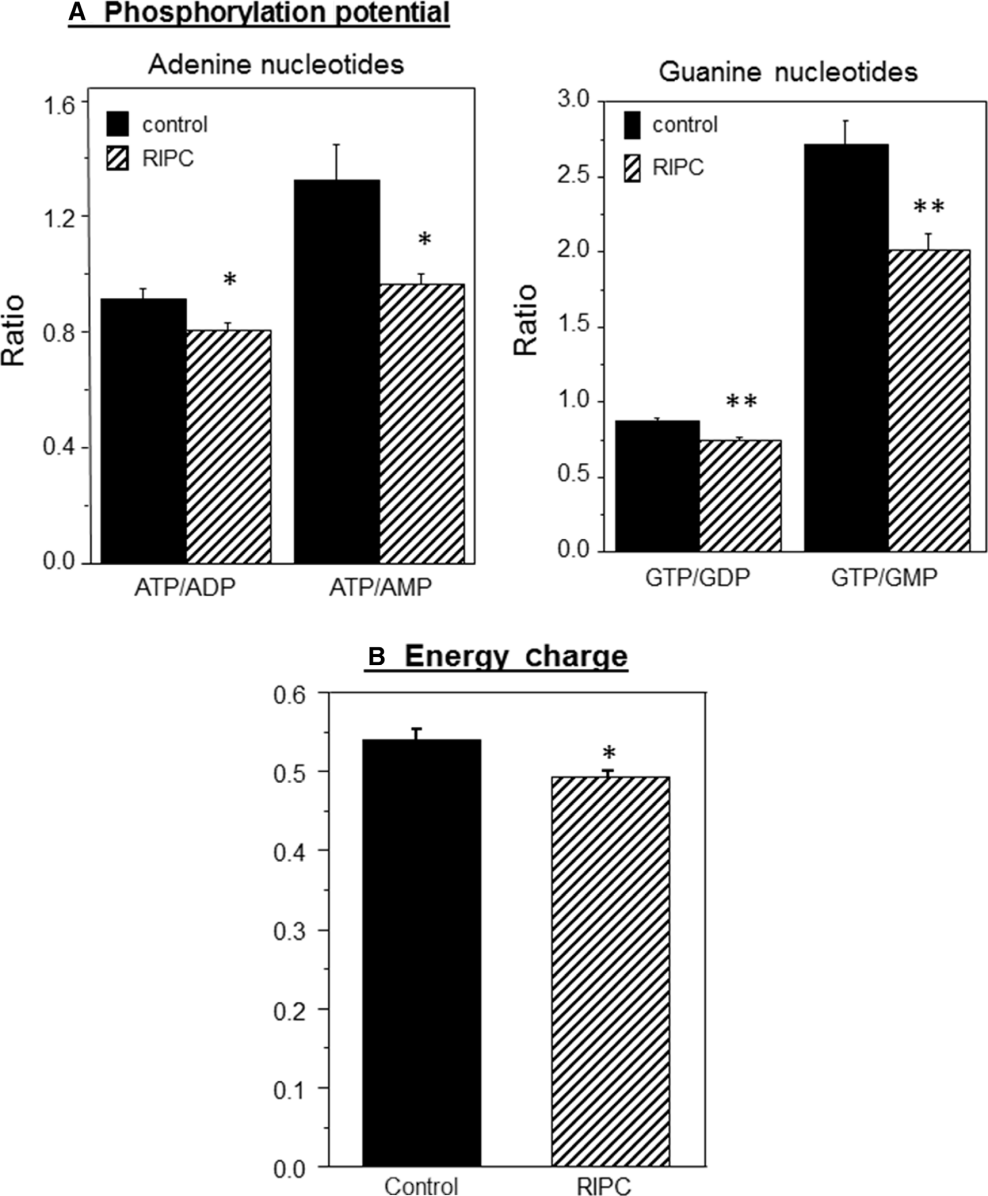
The effect of remote ischemic preconditioning (RIPC) on computed markers of ischemic stress. (A) Phosphorylation potential in hearts collected immediately after RIPC protocol. (B) Energy charge calculated using energy charge equation. Data are mean ± SEM (*n* = 7 hearts from each group). *P <* *0.05, **P <* *0.005.

With exception of adenosine (*P* < 0.02), none of the cellular metabolites measured in freshly excised hearts after 50 min of anesthesia with or without application of RIPC showed significant difference. Furthermore, upon calculating markers of ischemic stress (Atkinson and Walton 1967; Imura et al. 2011; Gout et al. 2014) including phosphorylation potential and adenylate energy charge, RIPC was found to have a significant effect (Fig. 4). RIPC was associated with a lower phosphorylation potential (ATP/ADP, ATP/AMP, GTP/GDP, and GTP/GMP) compared to control (Fig. 4A). Furthermore, adenylate energy charge, an index of the metabolic status and reflects the ability of the cell to make energy‐rich phosphates from precursors was also found to be lower in RIPC group compared to control (Fig. 4B). The energy charge was calculated using the following equation: Energy Charge = (0.5 ADP + ATP)/(AMP + ADP + ATP) (Gout et al. 2014).

#### The effect of RIPC on blood flow in hind limbs

**Figure 5:**
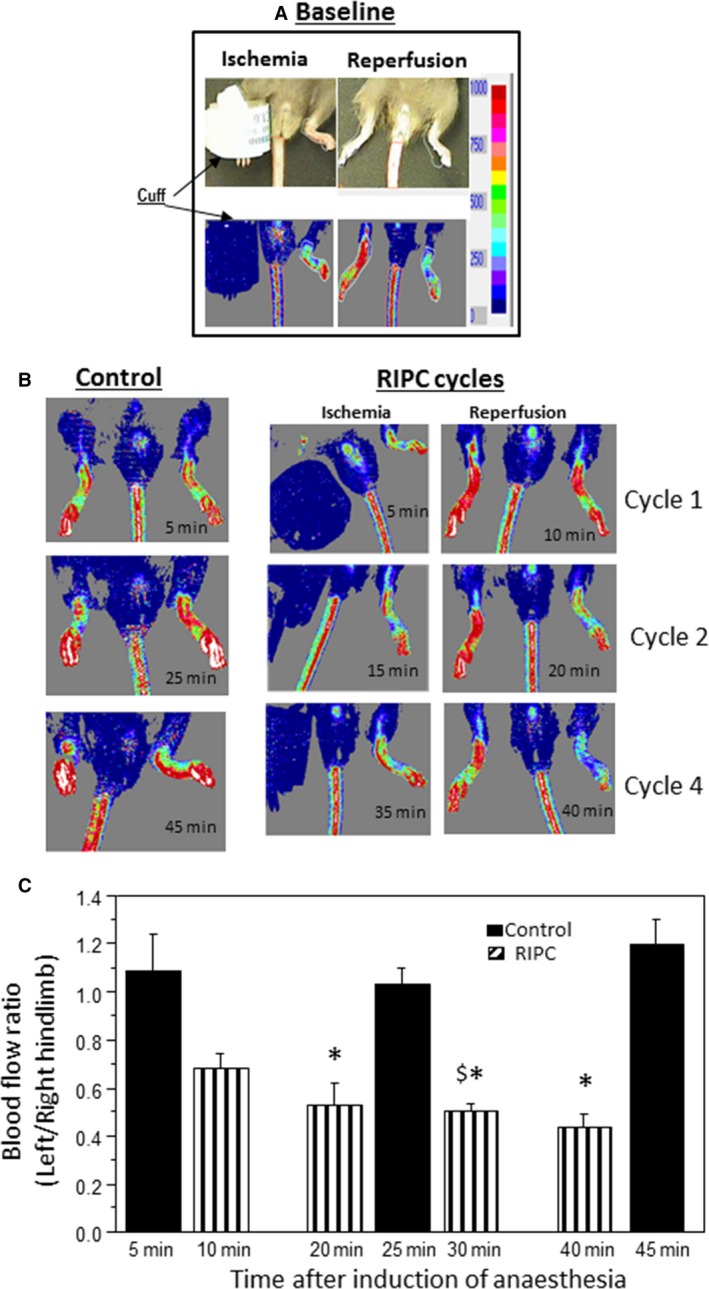
Blood flow ratio in hind limbs during remote ischemic preconditioning (RIPC) application. (A and B) Representative images showing changes of blood flow (color intensity) in both hind limbs of anesthetized mice. Top panel shows the flow during ischemia and following reperfusion (hyperemia). Lower left hand panel shows blood flow in anesthetized mouse overtime without application of RIPC. Lower right hand panel shows the changes in blood flow in limb exposed to four cycles of RIPC and the corresponding changes in its contralateral limb (white rectangles). (C) Changes in blood flow ratio expressed as left (uncuffed) limb over right in control mice or in mice exposed to RIPC application. Data extrapolated from color intensity (see colored panels above) and shown as mean ± SEM (*n* = 3 hearts for control and four for RIPC). Comparison was made between control and RIPC values at similar time points. *P <* *0.05 versus corresponding control values. ^$^ *P *<* *0.05 versus Cycle 1 (10 min).

Inflating blood pressure cuff during RIPC induced a complete block of the microcirculatory blood flow in the ischemic right limb (cuffed). During reperfusion there was a strong hyperemic (increased blood flow) response in this limb. However, this intervention in the right limb was associated with a sustained reduction in blood flow in the uncuffed nonischemic left limb (Fig. 5B). Figure 5C shows the calculated blood flow ratio for left limb/contralateral right limb. The ratio was ~1 in anesthetized control mice without RIPC. However, during RIPC application (reperfusion phase) the ratio was significantly lower compared to control mice. This appeared to be due to sustained drop in blood flow in the uncuffed hind limb.

## Discussion

### Validation of a mouse model of RIPC and protection against I/R

In this study we validated the use of mouse model of RIPC using a nonvolatile anesthetic where four cycles of I/R of hind limb resulted in significant cardioprotection as measured by size of infarction, cardiac enzyme release and developed tension (Fig. 2). Most RIPC studies use volatile anesthetics, which are known to produce pharmacological preconditioning (Ross and Foex 1999; Cromheecke et al. 2006). Other anesthetics including pentobarbital can affect plasma catecholamine which in turn may affect heart rate and other parameters including temperature and blood pressure (Baum et al. 1985). In our study we used tribromoethanol which is known to produce appropriate surgical anesthesia, with sufficient skeletal muscle relaxation and only a moderate degree of respiratory depression and has a good margin of safety (Murray et al. 2000). Furthermore, tribromoethanol has the most predictable and reproducible level of cardiac suppression and maintenance of heart rate compared to other anesthetics (Hart et al. 2001; Gao et al. 2011).

### Intermittent limb ischemia reduces blood flow in contralateral limb

During RIPC, the reduced blood flow in cuffed limb is associated with reactive hyperemia on reperfusion (Fig. 5A and B). However, blood flow in the uncuffed contralateral limb decreases during RIPC cycles (Fig. 5B and C). More importantly this vasoconstriction effect was dependent on the number of RIPC cycles as the first cycle had small effect while maximum effect appears following the third cycle. Most RIPC studies in mice used three or four cycles of 5 min ischemia and 5 min reperfusion (Lim et al. 2010; Cai et al. 2012; Kalakech et al. 2013). This interesting observation regarding flow in the uncuffed limb, suggests a gradual accumulative effect possibly due to release of vasoconstrictive endogenous factors during RIPC cycles. However, this observation does not appear to extend to other tissues as the blood flow in the tail was not altered (Fig. 5B). It is possible, however, that control of tail blood flow is different from that in limb flow and in view of the finding that the control/contribution of sympathetic‐mediated vasoconstriction responses in skeletal muscle is not fully elucidated (Thomas and Segal 2004).

Several studies have suggested release of humoral factors from the remotely preconditioned organ during RIPC which include adenosine, bradykinin, opioid, angiotensin 1, calcitonin gene‐related peptide, endocannabinoids, apolipoprotein A‐1, heat shock proteins, NO (Tanaka et al. 1998; Tang et al. 1999; Schoemaker and van Heijningen 2000; Xiao et al. 2001; Brzozowski et al. 2004a; Dong et al. 2004; Hibert et al. 2013), stromal cell‐derived factor‐1 (Davidson et al. 2013), and microRNA‐144 (Li et al. 2014). However, it is unlikely that factors involved in hyperemic response which include adenosine, bradykinin, and NO (Smits et al. 1995; Costa and Biaggioni 1998; Nyberg et al. 2010) are responsible for the observed vasoconstriction in the uncuffed limb (Fig. 5). One possible explanation for the vasoconstriction of microcirculatory blood vessels is that the release of local triggers in the preconditioned limb stimulate neural pathway(s) which in turn influence other organs (e.g., limb and heart). This is supported by studies showing limb ischemia induces norepinephrine release from the heart due to a systemic stress reaction, suggesting that this process involves adrenergic nerve termini of the heart (Oxman et al. 1997). The activation of neural pathways such as *α*1 and *α*2 adrenergic signaling pathways release epinephrine which leads to vasoconstriction of smooth muscle (Vatner et al. 1986). Moreover, *α*1‐adrenergic receptor has been implicated in the cardioprotection by RIPC (Taliyan et al. 2010). Whether measuring blood flow in our study is linked to changes in coronary circulation is not presently known. Nonetheless, it has been suggested that peripheral changes in skin blood flow can reflect generalized microvascular function including that of the coronary circulation (Khan et al. 2008).

In contrast to our study, a limited number of studies have reported vasodilatory effect when monitoring blood flow in selected remote organs (e.g., heart) during RIPC (Shimizu et al. 2007; Enko et al. 2011). In one study, RIPC application in conscious volunteers was found to induce vasodilatation of the contralateral artery (right brachial artery) (Enko et al. 2011) while in another, RIPC application in anesthetized pigs was associated with coronary vasodilation (Shimizu et al. 2007). These two studies were fundamentally different from our model where the volunteers were conscious and anesthetized pigs hearts were controlled by pacing during monitoring of coronary flow resistance (Shimizu et al. 2007).

It is evident from our data that intermittent limb ischemia triggers reduced blood flow in contralateral limb and that this effect appears to be dependent on number of cycles. The work suggests a possible role for neuronal (e.g., sympathetic) involvement. However, evidence for direct involvement of neural autonomic control can be extrapolated from monitoring changes in heart rate and HRV as discussed below.

### RIPC increases heart rate ex vivo and in vivo and alters *HRV*

During the characterization of this mouse RIPC model, we found that spontaneously beating Langendorff‐perfused hearts show significantly higher heart rate (computed from developed tension) compared to control hearts (Fig. 3A). This was also confirmed in vivo where % change from baseline was significantly different at the end of RIPC (50 min) compared to control (Fig. 3B). The relatively higher heart rate in isolated‐perfused heart after RIPC application could be triggered by different factors including changes in pacemaker cells excitability and the presence of neurotransmitter stores in the heart (Hoover et al. 2004; Tan et al. 2006). A likely trigger for increased heart rate is an activation of sympathetic activity. This is consistent with earlier evidence showing norepinephrine release from the heart following RIPC (Oxman et al. 1997) and evidence of PKA activation as shown by the increased phosphorylation of ryanodine receptor 2 (Abdul‐Ghani et al. 2013). *ß*‐blockers have been shown to block cardioprotection induced by spinal cord stimulation or peripheral surgical trauma (Southerland et al. 2007; Jones et al. 2009; Heusch et al. 2015). Finally very recent study suggests that cardioprotection by RIPC may involve intrinsic cardiac ganglia (Pickard et al. 2016).

Further evidence for the involvement of autonomic control of heart rate during RIPC comes from spectral analysis of HRV. HRV is widely used as a measure of ANS activity to the heart (sympatho‐vagal balance) (Roy and Ghatak 2013). The ratio between low and HF components of HRV spectra (LF/HF) ratio was relatively higher after reperfusion in the third cycle in RIPC group compared to control suggesting the involvement of sympathetic activity (Milicevic 2005). This finding contrasts with work showing intermittent human limb ischemia is associated with increased parasympathetic activity (Enko et al. 2011). This, however, was tested in conscious volunteers with no evidence of cardioprotection. The finding that RIPC is associated with increased in sympathetic activity can be used to explain the increase in heart rate and vasoconstriction in limb's blood vessel. Moreover, these activities would render the heart metabolically stressed. To address this issue, the myocardial energetics were monitored in control and in RIPC hearts.

### RIPC alters cardiac energetics

Unexpectedly, our study showed signs of mild ischemic stress in RIPC hearts as evidenced by a relatively lower myocardial ATP/ADP, ATP/AMP, GTP/GDP, and GTP/GMP ratios and in energy charge (Fig. 4). Clearly the extent of this anaerobic stress is relatively smaller than what is seen following classical preconditioning where ATP levels fall by as much as 30% (Murry et al. 1990). The importance/relevance of this mild stress is not presently known. It is likely, however, that this change could trigger signaling pathways involved in protecting the heart during RIPC.

## Summary and Conclusions

RIPC is associated with changes in cardiac and distal cardiovascular activities, consistent with ANS involvement which would contribute to cardioprotection.

## Clinical Perspective

Large amount of experimental data is available demonstrating that RIPC is a powerful, simple, noninvasive cardioprotective intervention. However, thus far this intervention has failed to convincingly translate into clinical practice. Lack of understanding of the signals mediating the response from distant organ to the heart or the cardiovascular changes during the application of RIPC and prior to index ischemia is likely to be key in hindering the translation of the RIPC intervention. Our work provides evidence for significant cardiovascular changes consistent with the involvement of ANS and myocardial metabolic stress during RIPC application which would provide an explanation for subsequent cardioprotective effects. These findings should prompt new translational/clinical studies that will take into account these changes in order to optimize conditions to take advantage of this intervention.

## Conflict of Interest

None declared.
